# MicroRNA miR-146a and further oncogenesis-related cellular microRNAs are dysregulated in HTLV-1-transformed T lymphocytes

**DOI:** 10.1186/1742-4690-5-100

**Published:** 2008-11-12

**Authors:** Klemens Pichler, Grit Schneider, Ralph Grassmann

**Affiliations:** 1 Institute of Clinical and Molecular Virology, University Erlangen-Nuremberg, Schlossgarten 4, Erlangen, Germany

## Abstract

**Background:**

Human T-lymphotropic virus type 1 (HTLV-1) is the etiologic agent of a severe and fatal lymphoproliferative disease of mainly CD4^+ ^T cell origin, adult T cell leukemia, which develops after prolonged viral persistence. Transformation of infected cells involves HTLV-1's oncoprotein Tax, which perturbs cell cycle regulation and modulates cellular gene expression. The latter function is also a hallmark of microRNAs, a rather new layer in the regulation of gene expression. Affecting e.g. proliferation, microRNAs constitute a potential target for viral interference on the way to persistence and transformation. Hence, we explored the interconnections between HTLV-1 and cellular microRNAs.

**Results:**

We report that several microRNAs – miRs 21, 24, 146a, 155 and 223 – are deregulated in HTLV-1-transformed cells. They are all upregulated except for miR-223, which is downregulated. Each of those microRNAs has ties to cancer. Their expression pattern forms a uniform phenotype among HTLV-transformed cells when compared to HTLV-negative control cells. In particular, miR-146a expression was found to be directly stimulated by Tax via NF-*κ*B-mediated transactivation of its promoter; a single NF-*κ*B site proximal to the transcription start point was necessary and sufficient for this to happen. An *in silico *analysis of potential target genes revealed candidates that might be coregulated by two or more of the aforementioned overexpressed microRNAs.

**Conclusion:**

These data demonstrate that cellular microRNAs are deregulated in HTLV-1-transformed T cells. In the case of miR-146a, this could be directly attributed to HTLV's oncoprotein Tax. Interference with cellular microRNAs may be crucial to maintaining persistence or may facilitate transformation of host cells.

## Background

Human T-lymphotropic virus type 1 (HTLV-1) is a *δ*-retrovirus infecting primarily CD4^+ ^T lymphocytes *in vivo*. Lifelong persistence ensues, which, after decades, can entail an aggressive neoplastic disease, adult T cell leukemia/lymphoma (ATLL). Another HTLV-1-associated disease presents as progressive neurodegeneration termed HTLV-associated myelopathy/tropical spastic paraparesis (HAM/TSP) [1-4]. HTLV's persistence manifests itself in T cell clones which remain detectable over many years even in non-leukemic infected individuals [5,6]. In the face of a continuous immune response this requires constant replenishment of infected cells. The virus achieves this through replication mainly in its provirus form, stimulation of cell division and, as a consequence, clonal amplification of infected cells.

HTLV-1 encodes accessory and regulatory proteins. While the accessory ones, p12, p30, p13 [7,8] and HBZ [9], are important for infectivity and viral replication [7,10], they are dispensable for immortalization [11-13]. The regulatory protein Tax drives viral mRNA synthesis by transactivating the HTLV-1 long terminal repeat promoter, Rex controls the synthesis of the structural proteins on a posttranscriptional level [14,15]. Both of them are essential for viral replication.

Tax confers the transforming properties on HTLV-1 [16]. It can immortalize T lymphocytes [17,18] and induce leukemia in transgenic mice [19]. Biochemically, several Tax functions, including transcriptional dysregulation and interference with cell cycle checkpoints, may contribute to its transforming capacity; they have been reviewed elsewhere [16]. For example, Tax is able to stimulate transcription by interacting with various signalling pathways. It activates both the canonical and the non-canonical pathways of nuclear factor kappa B (NF-*κ*B), the former by binding and stimulating IKK*γ*, a component of the inhibitor of kappa B kinase (IKK) complex [10]. Apart from NF-*κ*B, Tax is also capable of transactivating cellular promoters via direct contact with transcriptional activators CREB and SRF and with the coactivators p300/CBP [20,21].

Several publications describe phenotypical parallels between HTLV-transformed cells and regulatory T cells. These parallels comprise expression of markers like CD4, CD25, GITR [22] and FoxP3 [23,24]. However, it is still being disputed whether HTLV-transformed cells exhibit a distinct suppressive property [25,26]. When comparing HTLV-transformed cells with uninfected ones, looking at a phenotypically close population, i.e., one carrying the abovementioned markers, may help to obtain meaningful results. For this reason, we choose the phenotype of regulatory T cells as a starting point for our investigations into microRNA expression.

MicroRNAs have surfaced as being posttranscriptional regulators of gene expression [27]. The genes encoding them are transcribed by RNA polymerase II producing primary transcripts (pri-miR) which feature a stem-loop structure that is excised by an RNase, Drosha. The resulting hairpin is exported to the cytoplasm where another RNase, Dicer, converts it to the mature single-stranded microRNA [28]. The about 23 nucleotides long RNA molecules exert their function by binding to the 3' untranslated regions (3'-UTRs) of target mRNAs thus guiding a protein machinery, the microRNA-induced silencing complex (miRISC), which then suppresses translation of the mRNA. For in-depth reviews of microRNA function in lymphocytes see [29] and, with emphasis on microRNAs in virus infections, [30,31]. Cellular functions that microRNAs influence include lymphocyte differentiation [32,33], and some have even been implicated in oncogenesis [34,35].

To identify microRNAs involved in the pathogenesis of HTLV-associated disease, we selected a microRNA subset both characteristic of murine regulatory T cells (Treg) and reported to be deregulated in tumors. Within that subset, a single microRNA was downregulated and four microRNAs were overexpressed in HTLV-/Tax-transformed cell lines. Subsequent analysis established that one, miR-146a, was transactivated by Tax via promoter activation mediated by NF-*κ*B. Using online databases that catalogue predicted microRNA target genes we looked for instances of possible functional cooperation between the four overexpressed microRNAs.

## Results

### A text-mining approach identifies seven candidate microRNAs with potential for a part in HTLV pathogenesis

Since microRNAs affect cellular proliferation, differentiation and, ultimately, can play a part in tumorigenesis, we investigated their role in HTLV pathogenesis. Until now, no microRNAs encoded by HTLV-1 have been found although regulatory functions of non-coding HTLV-RNA have been described [36]. Consequently, using cellular microRNAs constitutes the only way for the virus to access that layer of regulation of gene expression. We chose a text-mining approach to narrow down the number of candidate microRNAs. This employed a set of two filters, first, one looking specifically at microRNAs expressed in naturally occurring T cell populations that exhibit closest similarity to cells transformed by HTLV and, second, another one selecting microRNAs – out of those returned by the first filter – with a documented link to oncogenesis.

Data suggest that regulatory T cells are the nearest phenotypical neighbour to CD4^+ ^T cells transformed by HTLV. The set of markers described for Treg comprises CD4, CD25, GITR, FoxP3 and 4-1BB, all of which have been found in HTLV-infected and/or -transformed cells [22-24,37]. FACS analyses confirmed this phenotype for the HTLV cell lines we used (data not shown). Cobb and colleagues compared microRNA expression patterns of regulatory and normal CD4^+ ^T cells, the latter with and without stimulation, in mice [38]. About 20 microRNAs were exclusively expressed or upregulated in Treg, out of those, seven had a published link to cancer: mir-21, miR-24, miR-146a, miR-155, miR-191, miR-214 and miR-223 [39]. All are described as being overexpressed in solid tumors or lymphoproliferative disease [39-41]; some have even been ascribed the potential to cause cancer [42]. Consequently, these microRNAs may contribute to transformation of HTLV-1-infected cells, i.e., the pathogenesis of ATLL, by either maintaining differentiation status or actively driving cells towards a transformed state.

### Upregulation of the BIC oncogene in HTLV-1-transformed lymphocytes can be seen on primary transcript level

Being the most prominent oncomiR, miR155, which is encoded by the *BIC *gene, was analyzed first. Originally, *BIC *was identified as an avian leukosis virus insertion site in B cell lymphomas of chicken [43] and, since then, has been verified as an oncogene in several experimental systems including transgenic mice [44]. The primary transcript, pri-miR-155, is generated by RNA polymerase II and processed to mature miR-155 afterwards. Its upregulated expression is also linked to human lymphoproliferative diseases like chronic lymphocytic leukemia, diffuse large B cell lymphoma and some forms of Burkitt's lymphoma [40,45,46].

**Figure 1:**
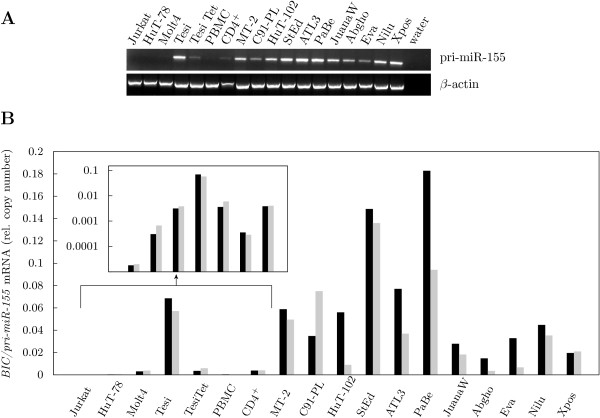
**Pre-miR-155 is uniformly expressed in HTLV-transformed lymphocytes**. (A) The primary transcript of the *BIC *gene, pri-miR-155, and *β*-actin (*ACTB*) mRNA were detected by RT-PCR. (B) Pri-miR-155 abundance was determined by qPCR. Relative copy number was computed by normalizing the pri-miR-155 transcripts to those of *ACTB*. Values of two independent measurements are shown.

To test whether elevated levels of *BIC *primary transcripts are a consistent feature of HTLV-infected cells RT-PCR was performed (Fig. 1A). RNA was isolated from cultures derived from ATLL patients (HuT-102, StEd, ATL3, PaBe, JuanaW), from HAM/TSP patients (Abgho, Eva, Nilu, Xpos) and from HTLV-1 and Tax *in vitro*-transformed cells (MT-2, C91-PL and Tesi, respectively). CD4^+ ^acute lymphoblastic leukemia (ALL) T cell lines, primary PBMC, and CD4^+ ^T cells from healthy donors served as HTLV-negative controls. HTLV-transformed cells uniformly expressed pri-miR-155 whereas it remained undetectable in other CD4^+ ^T cell leukemic cell lines (HuT-78, Jurkat, Molt4) (Fig. 1A). These results were in line with microarray data gleaned from Tesi cells, which strongly expressed *BIC *in the presence of Tax (Gene Expression Omnibus accession GSE10508, [37]).

Quantification of *BIC *transcripts in HTLV-infected cells by qPCR indicated high but variable RNA levels (Fig. 1B). Higher sensitivity allowed detection of pri-miR-155 transcripts in ALL cell lines Jurkat, HuT-78 and Molt4, which had been negative in normal RT-PCR, but even then transcript levels were very low (Fig. 1B, inset). Statistical analysis (Mann-Whitney U-test) revealed the increase of *BIC*/pri-miR-155 in HTLV-/Tax-transformed cells to be significant (*p *< 0.001).

### A set of Treg-specific mature microRNAs is upregulated in HTLV-1-transformed lymphocytes

**Figure 2:**
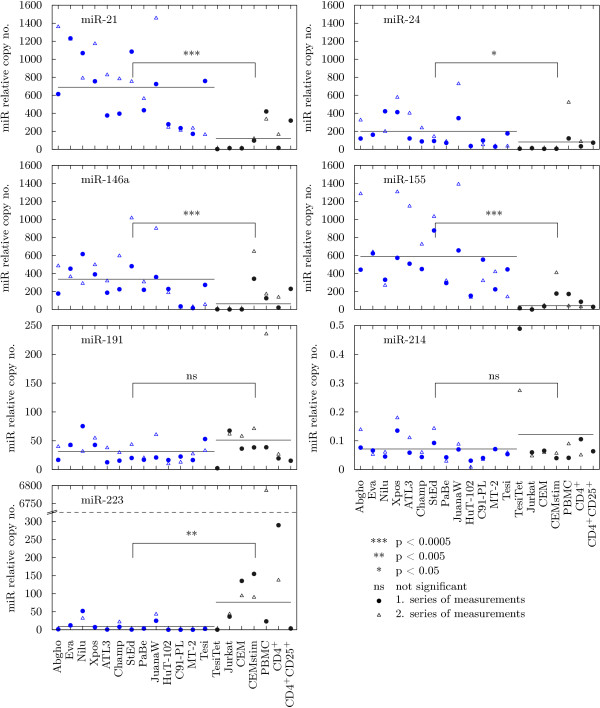
**OncomiRs are overexpressed in HTLV-transformed lymphocytes**. Expression levels of mature microRNAs miR-21, miR-24, miR-146a, miR-155, miR-191, miR-214 and miR-223 were detected by qPCR in two independent measurements. For each microRNA, samples from HTLV-/Tax-positive cell lines (blue) were compared to those of HTLV-/Tax-negative controls (black). U6 snRNA was used for normalization. Differences in the mean expression values were evaluated using the Mann-Whitney U-test.

Because, ultimately, the processed product of a microRNA gene exerts the gene's functions, levels of mature miR-21, miR-24, miR-146a, miR-155, miR-191, miR-214 and miR-223 were determined by qPCR (Fig. 2). This employed a specific stem-loop primer for reverse transcription which also elongated the miR reverse transcript to a length suitable for Taqman-based detection. Expression values were normalized to those of U6 small nuclear RNA (snRNA). The assay revealed high amounts of miR-155 in all HTLV-/Tax-positive cells with a mean relative copy number of 586 and, moreover, these expression levels were much higher than in HTLV-/Tax-negative controls, which had a mean value of only 41 (Fig. 2). The more than 14-fold difference turned out to be statistically highly significant (*p *≪ 0.0005). In summary, the observed upregulation of *BIC*/miR-155 suggests a benefit for HTLV-1 at some stage during its pathogenesis, i.e. miR-155 might be involved therein.

To complete the analysis of oncogenesis-related microRNAs, miR-21, miR-24, miR-146a, miR-191, miR-214 and miR-223 levels were determined in the same RNA samples used for miR-155 detection. The expression of miR-191 and miR-214 did not differ between HTLV-/Tax-positive and -negative cells (*p > *0.4 in both cases). Both showed only a moderate (miR-191) to low (miR-214) amount of mature product. When analyzing miR-223, an outlier value derived from one PBMC sample, which was more than 60 times higher than the mean of the rest of the values, biased expression in HTLV-negative samples towards a higher mean. However, even when ignoring that outlier, HTLV-negative cells expressed significantly more miR-223 than HTLV-positive ones (*p *≈ 0.01). Quantitative PCR revealed high expression of miRs 21, 24 and 146a in HTLV-/Tax-positive cells with mean values of 689, 200 and 335, respectively. In all three instances, expression significantly exceeded that of HTLV-negative controls. For all HTLV-1 positive cell lines the number of proviruses per cell was determined in qPCR analyses. A Spearman-Rho test, however, did not turn up any significant correlation between proviral load and microRNA expression level (see additional file 1: Table S1, Correlation analysis of provirus copy number and microRNA expression levels). Taken together, these results describe a characteristic pattern of oncogenesis-related microRNAs in HTLV-transformed lymphocytes: miRs 21, 24, 146a and 155 are upregulated, miR-223 is repressed and miRs 191 and 214 are unchanged compared to controls. The pattern, particulary the dysregulated microRNA species, might be relevant to the growth and survival of the transformed cell or might contribute to the process of transformation itself.

### Expression of endogenous miR-146a is stimulated by HTLV-1 Tax

**Figure 3:**
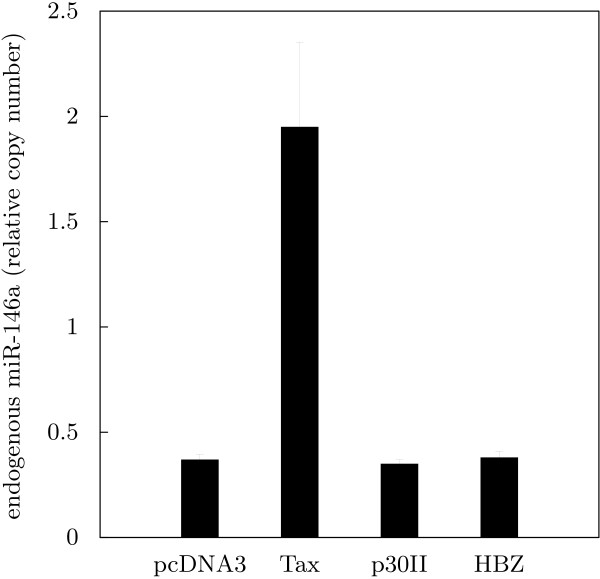
**Expression of endogenous miR-146a is stimulated by HTLV-1 Tax**. Jurkat T cells were transfected with expression plasmids for the HTLV-1 proteins Tax, p30^*II *^and HBZ or a control (pcDNA3). After 48 hours, RNA was extracted and subjected to qPCR detection of mature miR-146a. U6 snRNA was used for normalization. Values represent the means of at least three independent experiments.

The observed overexpression of miRs 21, 24, 146a and 155 raised the question whether this was due to viral interference. In particular, HTLV-1 Tax is a prime candidate for mediating such interference, but other viral proteins (p30^*II*^, HBZ) known to have an impact on cellular gene expression were also tested. We investigated the effect of ectopically expressed viral proteins on endogenous microRNAs in Jurkat T cells by transfecting them with expression plasmids for Tax, p30^*II *^and HBZ. After 48 hours, extracted RNA was assayed for the presence of mature microRNAs 21, 24, 146a and 155. Among the four microRNAs, one, miR-146a, was clearly upregulated in the Tax-expressing cells (Fig. 3). The observed difference with and without Tax was about 5-fold. Because suitable antibodies were not available, expression of HTLV-1 proteins p30^*II *^and HBZ could not be verified. Consequently, the lack of an effect on endogenous miR-146a might also be due to absence or too low expression levels of those proteins. This also applies to the other microRNAs, which were not significantly affected by any of the viral proteins (data not shown).

### MIRN146A promoter is transactivated by Tax via NF-*κ*B

**Figure 4:**
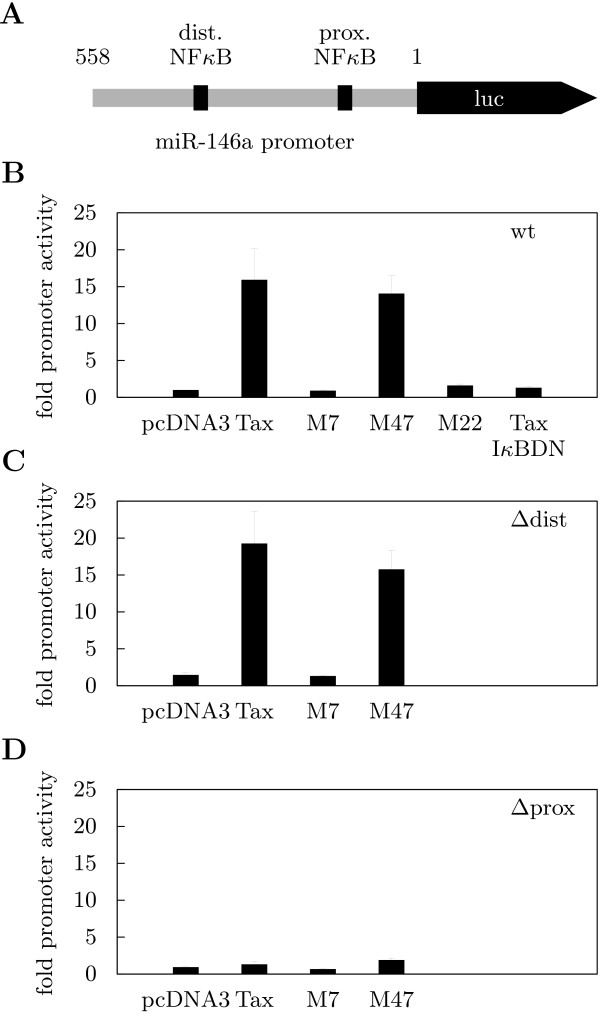
**MIRN146A (miR-146a) promoter is transactivated by HTLV-1 Tax through a single NF-*κ*B site**. (A) Schematic diagram of the 563 bp *MIRN146A *promoter sequence cloned into pGL3 basic. Proximal (prox.) NF-*κ*B binding site located at 68–77 bp, distal (dist.) at 386–395 bp. (B) Activity of the wildtype promoter was determined in reporter gene assays. Jurkat T cells were cotransfected with the reporter construct and expression plasmids for Tax, its mutant forms M7, M22 and M47, for a constitutively active NF-*κ*B inhibitor (I*κ*BDN) or a control (pcDNA3). Luciferase activity was normalized to protein content. Each combination was tested at least three times. (C), (D) Activity of the promoter with the distal (C) and proximal (D) NF-*κ*B site deleted. See (B) for experimental details. Tax mutant M22 and I BDN expression plasmids were not transfected.

We tested the hypothesis that the upregulation of miR-146a in the presence of Tax might happen through promoter transactivation. A 558 bp genomic fragment upstream of the miR-146a gene (*MIRN146A*) was cloned into a luciferase reporter plasmid and cotransfected into Jurkat T cells together with expression plasmids for viral Tax or controls (Fig. 4A). The cloned sequence contained two NF-*κ*B binding sites starting at positions 68 bp and 386 bp (MatInspector analysis and [47]). Wildtype Tax activated the promoter strongly (circa 15-fold) (Fig. 4B). To find involved transcriptional pathways, Tax mutants M7 (CREB^-^/NF-*κ*B^-^), M22 (CREB^+^/NF-*κ*B^-^) and M47 (CREB^-^/NF-*κ*B^+^) were tested in the reporter assay. Because M47 stimulated the promoter like wildtype Tax whereas M22 had no effect, this suggested NF-*κ*B-mediated transactivation. This conclusion was corroborated by cotransfecting a dominant active inhibitor of NF-*κ*B, I*κ*BDN, which completely suppressed the effect of wildtype Tax. Interestingly, deletion of the proximal NF-*κ*B binding site had the same effect (Fig. 3D), thus, indicating that this site was sufficient for controlling the promoter. Loss of the distal NF-*κ*B site did not affect promoter activity negatively (Fig. 3C). In short, HTLV-1 Tax specifically and strongly activated the *MIRN146A *promoter via single NF-*κ*B site proximal to the transcription start. This explained the elevated levels of mature miR-146a in HTLV-transformed lymphocytes.

### In silico analysis returns potential targets of collaborative microRNA effects

A bioinformatic approach to target genes could either emphasize the impact of each miR separately or focus on potential collaborative effects. Several online databases for target gene predictions are available, like microRNA.org [48], TargetScan [49], mirDB [50] and PicTar [51], yet only the microRNA.org resource allowed screening for genes targeted by several microRNAs simultaneously. After selecting that option, the resulting output was supplemented with predictions from the other databases (Table 1). For more than half the target genes predicted by microRNA.org, one or several binding sites for miRs 21, 24, 146a or 155 were predicted by other databases as well. An analysis of gene ontology terms (GO terms, [52]) revealed that most of the annotated genes impinge upon biological processes like signal transduction, regulation of cell proliferation and transcription.

**Table 1:** Genes with predicted simultaneous seed matches for miR-21, miR-24, miR-146a and miR-155.

Gene	Seed matches for miR				Seed matches in other databases	Gene ontology (GO) terms for biological processes
			
	21	24	146a	155		
*SH3TC2*	1	2	3	3	155^1^	signal transduction
*ITGB3*	3	2	3	1	-	cell adhesion
*MMP16*	2	1	2	3	24^1,2^, 146a^1^, 155^3^	-
*PURB*	2	2	2	2	21^1,2^, 24^1,2^	regulation of myeloid cell differentiation
*FAM130A1*	1	1	3	2	155^3^	positive regulation of transcription
*SMAD5*	2	1	3	1	-	signal transduction (TGF-*β*)
*ONECUT2*	2	2	1	2	146a^3^	organ morphogenesis
*DCX*	1	2	1	2	-	CNS development
*EIF2C4*	2	1	2	1	-	chromatin silencing
*CBFA2T2*	1	2	1	2	-	negative regulation of transcription
*JHDM1D*	3	1	1	1	21^3^	-
*SLC12A6*	1	1	1	2	24^1,2,3^, 155^3^	cell volume homeostasis
*ZNF532*	1	1	2	1	146a^1^	-
*TP53INP1*	2	1	1	1	24^1,2^, 155^1,2,3^	-
*KIAA1128*	2	1	1	1	-	-
*CLCN5*	2	2	1	1	24^1^	excretion
*FLJ25778*	1	1	2	1	-	-
*CREBL2*	1	1	1	2	21^1,2^, 24^1,2^, 146a^3^	signal transduction transcription
*RFXDC2*	1	1	1	2	146a^1,3^	-
*YOD1*	1	1	1	2	21^1^, 24^1^	amino acid metabolic process
*VGLL3*	2	1	2	1	24^3^	-
*PPM1D*	1	1	1	2	-	negative regulation of cell proliferation
*CYBB*	1	2	1	2	-	innate immune response, inflammatory resp
*RP11-93B10.1*	1	1	1	1	24^3^, 146a^1,2^, 155^1^	-
*COL19A1*	1	1	1	1	155^3^	-
*LOC348120*	1	1	1	1	-	-
*AFF1*	1	1	1	1	-	transcription
*RDX*	1	1	1	1	-	-
*RIT1*	1	1	1	1	21^1^, 24^1^, 155	signal transduction
*USP31*	1	1	1	1	-	-
*ARHGEF1*	1	1	1	1	21^3^	cell proliferation
*SERTAD2*	1	1	1	1	155^1^	negative regulation of cell growth

*In silico *analysis (microRNA.org) of genes carrying at least one binding site for each of the four microRNAs. Results were compared to those obtained from other databases (TargetScan, PicTar, mirDB) and hits listed in column "Seed matches in other databases".

^1^TargetScan, ^2^PicTar, ^3^mirDB

Importantly, the *in silico *analysis produced two genes already associated with regulation by microRNAs-*SMAD5*and *TP53INP1*. Both have been described to be repressed by miR-155 [53,54]. *TP53INP1 *is a proapoptotic protein involved in regulating p53-dependet cell death [55,56], *SMAD5 *is critical to TGF-*β*-mediated inhibition of proliferation in hematopoiesis [57]. Together with the upregulated expression of miR-21, miR24, miR-146a and miR-155, the conducted *in silico *analysis raises the possibility that, besides miR-155, other miRs might be involved in suppressing translation of *SMAD5 *or *TP53INP1 *even further. Experiments to test this hypothesis are currently ongoing. In summary, it might be worthwhile to re-examine genes which until now have only been tested for regulation by a single microRNA instead of the concerted effects of several.

## Discussion

MicroRNAs have emerged as an important factor in posttranscriptional regulation of gene expression with ties to cellular processes such as proliferation and differentiation. It has been demonstrated that microRNAs can contribute to the deregulation of such processes [34]. Consequently, the use of microRNAs may help a virus to attain e.g. persistence and, indeed, some viruses bring their own microRNAs [30,31]. Lacking any self-encoded microRNAs, HTLV could still interfere with host cell microRNAs in order to drive it towards accelerated proliferation and longevity.

This study approached HTLV-1's impact on cellular microRNA expression, focussing on a defined subset of oncogenesis-related specimens rather than relying on undirected microarray screening techniques. The selection procedure took into account (a) Treg-specific expression patterns of microRNAs and (b) available functional characterization, i.e., links to oncogenicity. While the patterns were based on data gathered in mice, comparability was maintained by phenotypically characterizing the cell lines used in this study (data not shown). This ensured continuity with both the mouse data and published descriptions of HTLV-transformed T cells' phenotype. Out of a panel of seven microRNAs, four were highly and significantly upregulated in HTLV-/Tax-positive samples as compared to controls, namely miR-21, miR-24, miR-146a and miR-155, whereas miR-223 was downregulated. The remaining two microRNAs, miR-191 and miR-214, were present in small amounts yet not regulated. Overall, the emerging pattern of microRNA expression in HTLV-transformed cells even within the investigated group of seven miRs illustrates the validity of our selective approach. Moreover, the pattern might contribute to a more detailed phenotypic characterization of HTLV-transformed cells, complementing well established protein markers. *BIC *and its mature product, miR-155, can be regarded as a prototypical oncomiR since it was first described as an integration site for avian leukosis virus before being identified as capapble of cooperation with myc in cancerogenesis [43]. Moreover, miR-155 appears to be involved in regulating a variety of lymphoid processes, particularly differentiation [58]. The overexpression of miR-155 in HTLV-transformed cell lines fits into that picture, hinting at a possible involvement in the pathogenesis of HTLV-associate disease at some point. Because the expression level of the *BIC *primary transcripts mirrored – insomuch as they were elevated – those of the mature miR-155, this indicated that miR-155 expression is probably controlled on a transcriptional level. Direct viral interference – in particular one of HTLV-1 Tax – with *BIC*/miR-155 expression could not be detected. The differences seen in Tesi cells, with and without Tax, could not be reproduced in an independent system. Clarifying this issue probably would have to take into account aspects of hematpoietic differentiation. It is worth mentioning that, though the association of hematologic malignancies with high miR-155 expression has been repeatedly described, no mechanism for its upregulation has been presented. Involvement of transcription factors AP-1 and NF-*κ*B during normal immune function of B cells has been described, however [59,60].

While expression levels of miR-191 and miR-214 did not significantly differ between HTLV-positive and -negative samples, miR-223 was downregulated in an HTLV context. The latter is particularly interesting since reduced miR-223 abundance has been described recently in hepatocellular carcinoma [61]. When overexpressed, miR-223 led to a decrease in cell viability by targeting stathmin. According to this link between miR-223 and stathmin, the observed downregulation of miR-223 in our study could potentially be involved in HTLV-1 cell-to cell spread *in vivo *[62,63].

Ectopically expressing HTLV-1 Tax in Jurkat T cells, which express only low levels of miR-146a, entailed a marked increase in miR-146a expression, thus, demonstrating that Tax is able to boost the cellular signals underlying that expression. Subsequent promoter analysis refined the initial observation, showing a circa 15-fold activation by Tax and explained the observed high levels of miR-146a in HTLV-transformed cell lines. Using mutated forms of Tax and the coexpression of a dominant active NF-*κ*B inhibitor, we were able to pinpoint the transactivation as being mediated via NF-*κ*B. These findings are in line with data by other groups who described NF-*κ*B regulation of miR-146a expression [47]. The previously published upregulation of miR-146a expression by EBV LMP-1 is of interest because it adds to the relevance of our findings [64,65]. Conceivably, this constitutes an important facet in both viruses' efforts to establish perstistence or their potential to bring about malignant transformation. In contrast to activation by LMP-1, Tax uses only one of the two NF-*κ*B sites present in the promoter sequence proximal to the transcriptional start site. Other transcription factors do not seem to participate in the Tax effect since the deletion of that aforementioned singular NF-*κ*B site completely abrogated promoter activity. Recent data by Bhaumik et al. describe a suppressive effect of miR-146a on NF-*κ*B signaling [66]. This could constitute a negative feedback loop of miR-146a on its own expression, which is rendered inoperative in an HTLV context. Taken together, our studies indicate that miR-146a stimulation in HTLV-transformed cells can be traced back to promoter transactivation by HTLV-1 Tax via NF-*κ*B.

Defining target genes for microRNAs bioinformatically is difficult owing to inherent inaccuracies in predictive algorithms, because, unlike siRNAs, microRNAs do not depend on perfect sequence complementarity to targets. Nevertheless, some targets have been described, like *IRAK6 *and *TRAF1 *3'-UTRs for miR-146a [47]. Another study [65] found endogenous *IRAK6 *and *TRAF1 *mRNA levels to be not or barely (respectively) miR-146a-regulated which could hint at the involvement of further regulatory elements, like additional microRNAs. Looking at cooperative effects of several microRNAs on a given mRNA might help avoiding such conflicting data. Interestingly, two targets ascribed to miR-155, *SMAD5 *[53] and *TP53INP1 *[54], came up in our analysis as having predicted binding sites for all four upregulated microRANs, miR-21, miR-24, miR-146a and miR-155. In the latter case, this was bolstered by congruent predictions from different databases. An upcoming paper describes that *TP53INP1 *is targeted by miRs 93 and 130b which were found to be overexpressed in both samples from patients suffering from acute ATLL and ATLL-derived cell lines [67]. The same paper also mentions the upregulation of miR-155 in ATLL patient samples. This strengthens our point that microRNAs do play a role in the pathogenesis of HTLV-associated disease. With regard to our findings, the data from Yeung at al. open up the possibility that a combined 'attack' of miR-155, miR-93, miR130b and maybe one the microRNAs investigated in this study on *TP53INP1 *might even increase documented microRNA effects on its mRNA. In summary, a combinatorial approach to the search for microRNA targets might help finding new targets or refining the regulation of known ones.

## Conclusion

This study analyzed the impact of HTLV-1 on cellular microRNAs. Filtering microRNAs for phenotypic restriction and oncogenesis-relatedness produced a set of seven microRNAs out of which four (miR-21, miR-24, miR-146a and miR-155) were overexpressed in HTLV-transformed cells. MicroRNA miR-223, however, was significantly repressed in an HTLV context. The validity of our approach is illustrated by the fact that it delivered miR-155, which appears to be a key player in lymphocyte malignancies, and miR-146a, whose upregulation has also been described in an EBV context [64,65]. The latter suggests this to be a more common phenomenon in the pathogenesis of persistent viruses, which should be inspected more closely. Moreover, taking into account possible collaborative effects of microRNAs when looking for target genes might reveal targets whose regulation is easily missed when testing a single microRNA or might refine our knowledge about regulation of known targets.

## Methods

### Cell culture

HTLV-1-negative acute lymphoblastic leukemia (ALL) T cell lines Jurkat, HuT-78, and CEM (CCRF-CEM), the ATLL patient-derived HTLV-1-positive T cell lines HuT-102, StEd, ATL-3, PaBe, JuanaW, Champ, and the HTLV-1 *in vitro*-immortalized, interleukin-2 (IL-2)-independent T cell lines C91-PL and MT-2 were kept as described [37]. The HAM/TSP patient-derived HTLV-1-positive T cell lines Abgho, Nilu (both 40 U/mL IL-2), Eva and Xpos (both 20 U/mL IL-2) were cultured in RPMI 1640 containing 40% Panserin (PAN-Biotech, Aidenbach, Germany), 20% fetal calf serum (FCS), glutamine (0.35 g/L), streptomycin and IL-2 (Roche Diagnostics, Mannheim, Germany) as indicated. The cell line Tesi was cultured as described [37]. Tesi is a Tax *in vitro*-immortalized T cell line featuring tetracycline-repressible Tax expression [18]; for complete Tax repression, cells were grown in medium containing 1 *μ*g/mL tetracycline for ten days. In order to stimulate CEM cells, 0.1 *μ*g/mL phorbolmyristate actetate (Sigma, Hamburg, Germany) and 2 *μ*M ionomycine (Calbiochem, San Diego, CA) were added to the medium for 18 hours.

### Isolation of PBMC and lymphocyte populations

Peripheral blood mononuclear cells (PBMC) were isolated from buffy coats from health donors (Institute of Transfusion Medicine, Suhl, Germany) by Ficoll density gradient (Biocoll, Biochrom, Berlin, Germany). CD4^+ ^and CD4^+^CD25^+ ^T cell subsets were separated from PBMC using the Regulatory T Cell Isolation kit (Miltenyi Biotech, Bergisch-Gladbach, Germany) and subjected to RNA extraction immediately afterwards.

### MicroRNA primary transcript and mRNA detection

Total cellular RNA from cell lines and PBMCs was isolated (Trizol, Invitrogen, Karlsruhe, Germany) and reversely transcribed (Superscript II, Invitrogen) using random hexamer primers (Invitrogen). Primers and probes are listed in additional file 2 (Table S2, Primers and probes). Quantitative real-time RT-PCR (qPCR) was performed on an ABI Prism 7700 Sequence Analyzer (Applied Biosystems, Foster City, CA) from 200 ng cDNA. In RT-PCR, 500 ng cDNA were used as template. Expression levels were computed by interpolation from standard curves generated from plasmids and calculating the mean of triplicate samples. *β*-actin (*ACTB*) was used for normalization.

### Quantification of mature microRNAs

Quantification of mature microRNAs was performed using TaqMan MicroRNA Assays (Applied Biosystems, Darmstadt, Germany) according to the manufacturer's protocol. Reverse transcription (RT) employed the MicroRNA Reverse Transcription Kit (Applied Biosystems, Darmstadt, Germany). Mature microRNAs were reversely transcribed using specific stem-loop primers which allow for generation of cDNA and, at the same time, elongation of the transcript up to a length amenable to analysis by qPCR. Input was 10 ng of total cellular RNA per RT reaction. After real time qPCR, expression values were normalized to that of U6 small nuclear RNA (RNU6B). For all of the HTLV-/Tax-positive cell lines as well as the PBMC, CD4 and one series of CEM (stim) cells, RNU44 transcripts were analyzed in parallel as an alternative normalization control. A comparison of U6, U44 and the geometric mean of both as normalization controls for microRNA expression is given in additional file 3 (Figure S3, Quantification and comparison of U44 expression in HTLV-1-/Tax-positive and -negative cells) and additional file 4 (Table S4, MicroRNA expression normalized to U6, U44 or U6/U44 geometric mean). Standard curves were generated using DNA oligos with sequences identical to those of the mature microRNAs. The quantification was conducted in two independent measurement series, each of which comprised separate RNA extraction, reverse transcription and subsequent qPCR.

### Determination of provirus copy numbers

Proviral copy numbers were determined according to Dehee et al. [68]. Briefly, genomic DNA was extracted from cells using Trizol (Invitrogen) and 100 ng were used per qPCR reaction. In contrast to [68], the total volume reaction volume was 20 *μ*L each and the annealing/extension cycle was one minute at 60 degrees celsius. Primers SK110 and SK111, which are located in the *pol *region of the HTLV-1 genome, detected provirus copies. Values were normalized to copies of the human albumin gene (*ALB*), of which two alleles per cells are present. Standard curves were generated from a plasmid, pcHTLV-ALB, carrying both relevant target sequences of the qPCR.

### Analysis of effects of HTLV-1 proteins on endogenous microRNAs

Jurkat T cells were transfected by electroporation with expression plasmids for HTLV-1 Tax, p30^*II *^[69] or HBZ [13]. After 48 hours, RNA was extracted and the level of microRNAs determined as described in section 'Quantification of mature microRNAs'.

### Promoter analysis

A 558 bp fragment of genomic DNA [Genbank: NT_023133.12 Hs5_23289: 4704215-4704772] upstream of the *MIRNA146A *(miR-146a) gene, located on chromosome 5q33.3, was cloned into pGL3basic (Promega, Mannheim, Germany) using *Pwo *polymerase (Roche Diagnostics) and primers containing *Nhe*I and *Hin*dIII restriction sites producing pGL3basic:miR146aprom. The NF-*κ*B deletion mutants pGL3basic:miR146aΔNF-*κ*Bdist and pGL3basic:miR146aΔNF-*κ*Bprox were generated by overlap-extension PCR. All plasmids were sequenced. Tax impact on the miR-146a promoter was tested in reporter gene assays in transfected Jurkat T cells as described [37]. Briefly, Jurkat T cells were cotransfected by electroporation (EasyJect plus, Equibio, Ashford, United Kingdom, at 290 V and 1500 *μ*F) with 20 *μ*g of the reporter construct and either 20 *μ*g pcDNA3, pcTax [70], pM7, pM22, pM47 [71] or 2 *μ*g pI*κ*BDN [72]. Luciferase activity was measured after 48 hours (Orion Microplate Luminometer; Berthold, Pforzheim, Germany) and normalized to protein concentration. Each combination was tested at least three times. Values are given as multiple of the control (pGL3basic:miR-146aprom plus pcDNA3).

### Statistical analysis

For evaluation of differences in expression levels of HTLV-1-/Tax-positive and -negative samples, the Mann-Whitney U-test was applied using SPSS version 12.0.2 (SPSS, Chicago, IL). Stimulated CEM cells were excluded from the calculation. Analysis of correlations between provirus copy number and microRNA expression employed the Spearman-Rho test.

## Competing interests

The authors declare that they have no competing interests.

## Authors' contributions

KP carried out quantification of RNAs and luciferase assays, performed the statistical and database analyses and wrote the manuscript. GS carried out luciferase assays. RG conceived of the study and participated in its design and coordination. All authors read and approved the final manuscript.

## Supplementary Material

Additional file 1 **Correlation analysis of provirus copy number and microRNA expression levels**. Provirus copy number (PL) in seven cell lines (Eva, Xpos, StEd, PaBe, JuaW, C91-PL, MT-2) was determined as described in materials and methods. Correlations between PL and microRNA expression levels were then evaluated using the Spearman-Rho test. Resulting correlation coefficients, *P *values and the number of analyzed samples are given in the table. Click here for file

Additional file 2 **Primers and probes**. Supplementary table S1 lists primers used for cloning miR-146a promoter and its NF-*κ*B deletion mutants. Furthermore, primers and probes used in RT-PCR and qPCR analyses are given unless obtained from commercial sources. Click here for file

Additional file 3 **Quantification and comparison of U44 expression in HTLV-1-/Tax-positive and -negative cells**. In addition to RNU6B (U6) transcripts, RNU44 (U44) was quantified in the samples indicated. Quantification was performed as described in materials and methods. To assess variation of expression values, i.e., the usefulness of U6 and U44 as normalization controls, the mean and coefficient of variation (CV) was determined. The CV was calculated as standard deviation devided by the mean. Smaller CV values indicate higher overall expression stability. Click here for file

Additional file 4 **MicroRNA expression normalized to U6, U44 or U6/U44 geometric mean**. MicroRNA expression was normalized to both U6, U44 and their geometric mean in the following samples: Abgho, Nilu, Eva, Xpos, ATL-3, JuaW, StEd, Champ, PaBe, HuT-102, C91-PL, MT-2, CEM, PBMC and CD4^+ ^T cells. Afterwards, differences in expression levels in HTLV-/Tax-positive vs. -negative cells was evaluated using the Mann-Whitney test. Note that the sample set is not identical to the one in Figure 2 and, therefore, results may differ. Click here for file
