# Supplementary material for: MicroRNA miR-146a and further oncogenesis-related cellular microRNAs are dysregulated in HTLV-1-transformed T lymphocytes
Source: Retrovirology. 2008 Nov 12;5:100. doi: 10.1186/1742-4690-5-100 (PMC2628945; doi:10.1186/1742-4690-5-100)
Supplement: Additional file 1 — Correlation analysis of provirus copy number and microRNA expression levels. Provirus copy number (PL) in seven cell lines (Eva, Xpos, StEd, PaBe, JuaW, C91-PL, MT-2) was determined as described in materials and methods. Correlations between PL and microRNA expression levels were then evaluated using the Spearman-Rho test. Resulting correlation coefficients, P values and the number of analyzed samples are given in the table. [file 1742-4690-5-100-S1.pdf]

SUPPLEMENTARY TABLE S1

provirus copy number per cell (PL)							
cell line	C91-PL	MT-2	JuaW	PaBe	StEd	Eva	Xpos
PL	.96	1.99	.33	.28	1.57	.44	.40

	Correlation PL – microRNA expression		
microRNA	correlation coefficient	*P*	*N*
miR-223	−.643	.119	7
miR-214	.321	.482	7
miR-191	−.214	.645	7
miR-155	−.071	.879	7
miR-146a	−.107	.819	7
miR-24	−.429	.337	7
miR-21	−.179	.702	7
