# Supplementary material for: MicroRNA miR-146a and further oncogenesis-related cellular microRNAs are dysregulated in HTLV-1-transformed T lymphocytes
Source: Retrovirology. 2008 Nov 12;5:100. doi: 10.1186/1742-4690-5-100 (PMC2628945; doi:10.1186/1742-4690-5-100)
Supplement: Additional file 2 — Primers and probes. Supplementary table S1 lists primers used for cloning miR-146a promoter and its NF-κB deletion mutants. Furthermore, primers and probes used in RT-PCR and qPCR analyses are given unless obtained from commercial sources. [file 1742-4690-5-100-S2.pdf]

Supplementary table S2:

Primers and probes	
	BIC transcript RT-PCR
pri-miR-155 fwd	TATAAAGCTTCTAATGGTGGCACAAACCAG
pri-miR-155 rev	TTAATCTAGAAAACCTGCAATTAAGAATGACATG
	BIC transcript qPCR
pri-miR-155 TaqMan fwd	TCAAGAACAACCTACCAGAGACCTT
pri-miR-155 TaqMan rev	TCCTGGTTTGTGCCACCAT
pri-miR-155 TaqMan probe	ACCTTGGCTCTCCCACCCAATGGA
	ACTB qPCR
ACTB TaqMan fwd	CCTCGCCTTTGCCGA
ACTB TaqMan rev	TGGTGCCTGGGGCG
ACTB TaqMan probe	CCGCCGCCCGTCCACACCCGCC
	miR146a promoter cloning
146a_prom_fwd	AATATAGCTAGCTTCTGCCTGATCTTCTCC
146a_prom_rev	ATATACTCGAGAGAGGAAGGCAGCTAAGG
146a_promoter_delta_dist_fwd	CGATAAAGCTCTCCGCGGGGCTGCG
146a_promoter_delta_dist_rev	CGCAGCCCCGCGGAGAGCTTTATCG
146a_promoter_delta_prox_fwd	GAGGGATCTAGAAAGAGAGGGTTAGCG
146a_promoter_delta_prox_rev	CGCTAACCCTCTCTTTCTAGATCCCTC
