# Supplementary figures and images for: MicroRNA miR-146a and further oncogenesis-related cellular microRNAs are dysregulated in HTLV-1-transformed T lymphocytes
Source: Retrovirology. 2008 Nov 12;5:100. doi: 10.1186/1742-4690-5-100 (PMC2628945; doi:10.1186/1742-4690-5-100)

SUPPLEMENTARY FIGURE S3

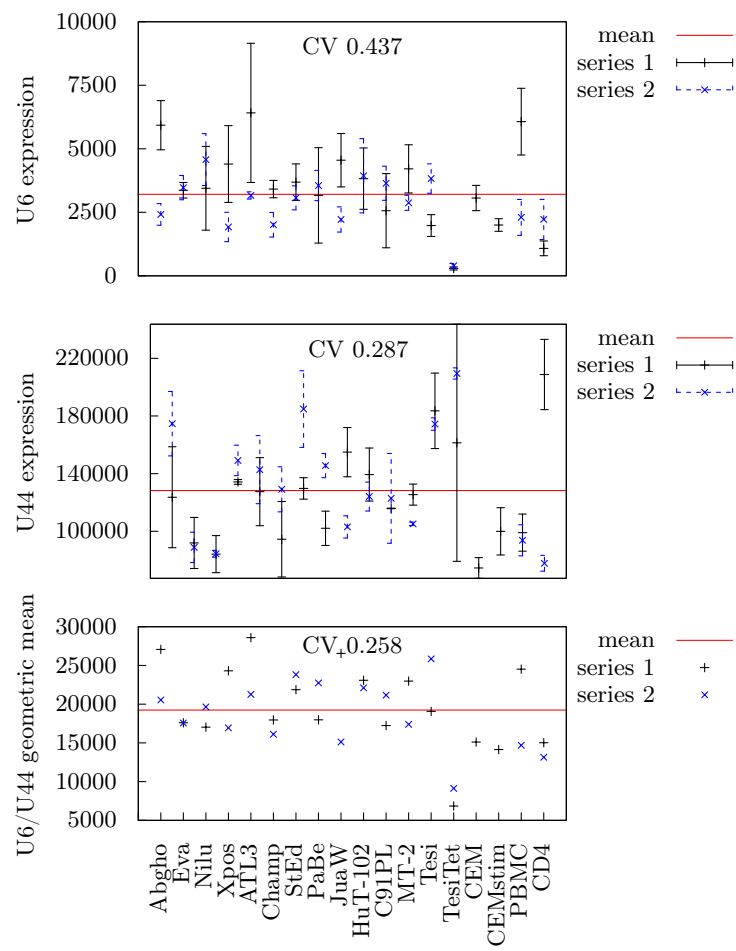

Supplement: Additional file 3 — Quantification and comparison of U44 expression in HTLV-1-/Tax-positive and -negative cells. In addition to RNU6B (U6) transcripts, RNU44 (U44) was quantified in the samples indicated. Quantification was performed as described in materials and methods. To assess variation of expression values, i.e., the usefulness of U6 and U44 as normalization controls, the mean and coefficient of variation (CV) was determined. The CV was calculated as standard deviation devided by the mean. Smaller CV values indicate higher overall expression stability. [file 1742-4690-5-100-S3.pdf]
