# Supplementary material for: MicroRNA miR-146a and further oncogenesis-related cellular microRNAs are dysregulated in HTLV-1-transformed T lymphocytes
Source: Retrovirology. 2008 Nov 12;5:100. doi: 10.1186/1742-4690-5-100 (PMC2628945; doi:10.1186/1742-4690-5-100)
Supplement: Additional file 4 — MicroRNA expression normalized to U6, U44 or U6/U44 geometric mean. MicroRNA expression was normalized to both U6, U44 and their geometric mean in the following samples: Abgho, Nilu, Eva, Xpos, ATL-3, JuaW, StEd, Champ, PaBe, HuT-102, C91-PL, MT-2, CEM, PBMC and CD4+ T cells. Afterwards, differences in expression levels in HTLV-/Tax-positive vs. -negative cells was evaluated using the Mann-Whitney test. Note that the sample set is not identical to the one in Figure 2 and, therefore, results may differ. [file 1742-4690-5-100-S4.pdf]

SUPPLEMENTARY TABLE S4

	(normalizing transcript).(microRNA)						
	u44.223	u44.214	u44.191	u44.155	u44.146a	u44.24	u44.21
Mann-Whitney	54	74	91	8	25	54	28
Z score	-.1629	-.749	0.000	-3.655	-2.906	-1.629	-2.774
P (asymp.)	.103	.454	1.000	.000	.004	.103	.006
P (exact)	.109	.476	1.000	.000	.002	.109	.004
	u6.223	u6.214	u6.191	u6.155	u6.146a	u6.24	u6.21
Mann-Whitney	44	62	79	3	16	45	17
Z score	-2.070	-1.277	-.528	-3.875	-3.303	-2.026	-3.259
P (asymp.)	.038	.202	.597	.000	.001	.043	.001
P (exact)	.039	.215	.620	.000	.000	.043	.000
	geo.223	geo.214	geo.191	geo.155	geo.146a	geo.24	geo.21
Mann-Whitney	45	79	85	4	16	48	22
Z score	-2.026	-.528	-.265	-3.831	-3.303	-1.894	-3.039
P (asymp.)	.043	.597	.792	.000	.001	.058	.002
P (exact)	.043	.620	.813	.000	.000	.060	.001
